# Health equity in Somalia? An evaluation of the progress made from 2006 to 2019 in reducing inequities in maternal and newborn health

**DOI:** 10.1186/s12939-023-02092-1

**Published:** 2024-03-05

**Authors:** Joana Morrison, Sk Md Mamunur Rahman Malik

**Affiliations:** 1 World Health Organization, Barcelona, Spain; 2 World Health Organization, Mogadishu, Somalia

**Keywords:** Somalia, Health inequities, Infant mortality, Contraceptive agents, Pregnancy in adolescence, Prenatal care, Midwifery, Breastfeeding

## Abstract

**Background:**

Every human being has the right to affordable, high-quality health services. However, mothers and children in wealthier households worldwide have better access to healthcare and lower mortality rates than those in lower-income ones. Despite Somalia’s fragile health system and the under-5 mortality rate being among the highest worldwide, it has made progress in increasing reproductive, maternal, and child health care coverage. However, evidence suggests that not all groups have benefited equally. We analysed secondary 2006 and 2018–19 data to monitor disparities in reproductive, maternal, and child health care in Somalia.

**Methods:**

The study’s variables of interest are the percentage of contraceptive prevalence through modern methods, adolescent fertility rate, prenatal care, the rate of births attended by midwives, the rate of births in a health care facility, the rate of early initiation of breastfeeding, stunting and wasting prevalence and care-seeking for children under-five. As the outcome variable, we analysed the under-five mortality rate. Using reliable data from secondary sources, we calculated the difference and ratio of the best and worst-performing groups for 2006 and 2018–19 in Somalia and measured the changes between the two.

**Results:**

Between 2006 and 2018–19, An increase in the difference between women with high and low incomes was noticed in terms of attended labours. Little change was noted regarding socioeconomic inequities in breastfeeding. The difference in the stunting prevalence between the highest and lowest income children decreased by 20.5 points, and the difference in the wasting prevalence of the highest and the lowest income children decreased by 9% points. Care-seeking increased by 31.1% points. Finally, although under-five mortality rates have decreased in the study period, a marked income slope remains.

**Conclusions:**

The study's findings indicate that Somalia achieved significant progress in reducing malnutrition inequalities in children, a positive development that may have also contributed to the decrease in under-five mortality rate inequities also reported in this study. However, an increase in inequalities related to access to contraception and healthcare for mothers is shown, as well as for care-seeking for sick children under the age of five. To ensure that all mothers and children have equal access to healthcare, it is crucial to enhance efforts in providing essential quality healthcare services and distributing them fairly and equitably across Somalia.

## Background

The number and rate of deaths of children under the age of 5 halved between 2000 and 2021 worldwide [1, 2]; however, it is well documented that the reduction has not been equal across global regions [3, 4]. Sub-Saharan Africa remains the region with the highest under-five mortality rate (U5MR), 72 deaths per 1000 live births– almost twice the global rate [1, 2]. Within regions, there are also inequalities in child health. The under-5 mortality rate in Somalia is among the highest worldwide (117 deaths per 1000 live births in 2021) [1, 2]. This is three times higher than the global average of 38 per 1000 live births. Compared to high-income countries, where the average is 5 deaths per 1000 live births, Somalia’s under-five mortality rate is 22 times higher [1, 2].

Some 287,000 women lost their lives in 2020 due to preventable causes related to pregnancy and childbirth [5, 6]. These resulted in a maternal mortality ratio (MMR) of 223 per 100,000 live births. Somalia is still one of the most dangerous places for women to give birth, with a maternal mortality ratio of 621 deaths per 100,000 live births, which is among the highest in the world [5]. This figure is well above the sustainable development goal (SDG) target of less than 70 per 100,000 live births [6].

There are still significant inequalities in access to opportunities for mothers and their children to live long and healthy lives based on where they live [7, 8]. To our knowledge, no studies have examined Somalia’s progress in addressing health inequalities despite indications that the country is not making sufficient strides towards meeting SDG3 targets [9]. The SDG principle of ‘Leave no one behind’ refers to achieving sustainable progress with equity. For this purpose, disaggregated analyses of available indicators are necessary for monitoring health equity to ensure the entire population benefits from progress towards SDG 3 indicators [10, 11].

This study aims to determine whether improvements in maternal and child health and outcomes are reaching all members of the population equally in Somalia, including those who are difficult to reach and in great need. We examine whether health inequalities in Somalia are decreasing despite the limited evidence and data available. Specifically, the study focuses on analysing absolute and relative inequalities in health variables in Somalia. It also examines how these inequalities have changed from 2006 to 2018–19.

## Methods

To achieve our objective of quantifying inequality in Somalia’s reproductive, maternal and child health (RMCH) indicators and monitoring these over time, we calculated the difference between disaggregated summary measures for 2006 and 2018–19.

### Data sources

This study utilises secondary data from two different surveys. The last Multiple Indicator Cluster Survey (MICS) conducted in Somalia took place in 2011 and was separated into two surveys, one for the Northeast Zone and another for Somaliland. Therefore, we were not able to compare results with the previous 2006 Somalia MICS. We accessed 2018–19 data from the Somali Health and Demographic Survey (SHDS) 2020 report [12] to compare inequality levels with 2006 and include the most recent data on disparities in RMCH in Somalia.

#### Multiple Indicator Cluster Survey 2006 Somalia

We obtained the 2006 Somali data from WHO’s Health Equity Assessment Toolkit (HEAT), a free open-source online software [13]. From HEAT, we downloaded UNICEF’s MICS 2006 Somalia data, which was re-analysed by the WHO Collaborating Center: The International Center for Equity in Health, within the Federal University of Pelotas [14]. The 2006 MICS survey sampling design followed a four-stage sample approach. It covered a population of women aged 15–49 and children under five from a list of households obtained from the 2005–2006 United Nations Development Programme Settlement Survey and WHO vaccination campaign data. The fieldwork consisted of interviewing 5969 households with a 99.5% response rate [15]. Data collection occurred during August and September 2006.

#### Somali Health and Demographic Survey 2018–19

The SHDS report was published in 2020. The data were collected 1 to 2 years prior (2018–19) [12]. The SHDS followed a three-stage stratified cluster sample design; 15,761 households were interviewed, with a 99% response rate. The response rate was similar throughout all three places of residence (urban, rural, and nomadic). The SHDS 2020 interviewed 16,715 women—11,884 were ever-married, and 4,831 were never married [12].

#### Variables: health indicators

This article evaluates the advancements in decreasing health inequalities for mothers and children under five in Somalia from 2005 to 2019. Because of the severe lack of good quality representative data on health inequalities in reproductive, mother and child health in Somalia, we had to narrow down our selection of variables relating to essential SDG–3 indicators. Our inclusion criteria were guided by availability and comparability with other countries in sub-Saharan Africa (SSA) and within Somalia. We utilised data that would permit conducting comparisons across time. The variables included in this study had to meet the criteria of sound methodology and sufficient sample size. The scope was narrowed only to include RMCH variables available for both datasets (MICS 2006 and SDHS 2020) that shared the same definition, fieldwork and methodology with minimal variation.

We analysed the following study variables: the percentage of contraceptive prevalence through traditional and modern methods, adolescent fertility rate (measured by births per 1000 women aged 15–19), antenatal care (at least one visit), births attended by experienced health personnel, the rate of births attended in a health care facility (%). Early initiation of breastfeeding (measured by infants breastfed within one hour of birth), care-seeking for children under the age of 5, the prevalence of stunting of children under five (%), and the prevalence of wasting and severe wasting of children under five (%). The outcome variable in this study was the under-five mortality rate in Somalia, measured by deaths per 1000 live births.

#### Inequality dimensions

Data disaggregation is recommended for health inequality monitoring and is encapsulated in the SDGs [16]. The variables in this study are disaggregated by wealth quintile (based on the income of the household head), educational level of the mother, and place of residence (urban, rural, or nomadic) for the MICS 2006 and SHDS 2020 [17, 18]. We included these levels of disaggregation in this study as wealth is a determining factor in healthcare access, and education is linked to health literacy and a person’s ability to make informed health decisions. A person’s place of residence, whether urban or rural, can significantly impact health outcomes due to differences in access to healthcare facilities and other services and resources relevant to health [19].

Data categories were comparable between surveys, and only data disaggregation by education needed standardisation. Somalia MICS 2006 used three educational categories, and the SHDS 2020 used four. To overcome this, we computed the difference and ratio between ‘no education’ and ‘secondary education’ for both surveys to establish changes in absolute and relative educational inequalities in RMCH. Data on nomadic populations were available from the SHDS; however, these were not included in this study as we could not access data on health inequalities for nomadic populations from the MICS survey or compare it. RMCH data disaggregated by whether populations were internally displaced (IDP) [17] were unavailable from both surveys and, therefore, were not included in this study as such.

### Analysis

#### Summary measures

We computed the difference between the most extreme values for each social variable category to determine absolute inequities as the difference between two population subgroups:

$$D\,=\,yhigh\, - \,ylow$$

We calculated the difference between rates among the highest and the lowest wealth quintiles for absolute economic inequities. For absolute educational inequality, we calculated the difference in rates for those with secondary education and those without qualifications. For absolute inequities by place of residence we computed the difference in rates between families living in urban households and those living in rural ones. 

To determine relative inequalities, we calculated the highest to the lowest wealth quintiles ratio, the highest to the lowest educational level ratio, and the ratio of urban and rural populations for data extracted from the MICS 2006 and SDHDS 2020. Relative inequalities were calculated as the ratio of the two groups on each end of the range and is on a logarithmic scale:

$$R\,=\,yhighest\,wealth\,quintile\,/\,ylowest$$

We computed this study’s summary measures (difference and ratio) using the WHO Health Equity Assessment Toolkit and Microsoft Excel.

We then calculated the difference between the MICS 2006 and SDHDS 2020 summary measures (difference and ratio) to determine whether absolute and relative inequality measures changed: whether these increased, decreased, or stayed the same.

## Results

### Contraceptive methods and modern contraception use

In Somalia, from 2006 to 2018–2019, the most significant change in contraception use was seen in the ratio of women in the highest to the lowest income groups (increased by 21.2% points). For modern contraception, the same is observed. The ratio of high-income to low-income women using modern contraceptives increased by 28.5% points from 2006 to 2918–19.

### Adolescent fertility rates

Between 2006 and 2018–2019, the difference between the highest and lowest income quintiles in the adolescent fertility rate decreased by 23.6 per 1000 for economic status, 113.3 per 1000 for education, and 32 per 1000 for place of residence. The most significant change was a decrease in the difference between adolescent fertility rates among those with the highest educational attainment and those with the lowest. During this same time range, the ratio of adolescent fertility rates of women in the highest to women in the lowest income quintiles increased by 0.86 per 1000. Between women with secondary and no education, the ratio in the adolescent fertility rate decreased by 4.21 per 1,000 and place of residence by 0.03 per 1,000.

### Prenatal care

Between 2006 and 2018–19, there was an increase of 39.2% points in the difference between the women with the highest and lowest income levels in terms of the percentage who attended at least four prenatal visits during pregnancy. The difference increased by 45.3% points between women who had completed secondary school and women without formal education. It increased by 10.4% points between women living in urban and rural settings (Table 1).

**Table 1:** The percentage point difference between the best and worst performing group in each determinant category (economic status, education, place of residence) for each inequality indicator for the use of reproductive, maternal, and newborn care in Somalia in 2006 and 2018–19

Exposure indicators	Determinant	Measure	2006	2018–19	Change from 2006 to 2018–19 (percentage points)	Direction
Contraception use (%)	Economic status (wealth quintiles)	Difference (percentage points)	6.9	8.7	1.8	Increase
		Ratio (percentage points)	1.6	22.75	21.15	Increase
	Education (no studies, primary, secondary)	Difference (percentage points)	8.4	17.1	8.7	Increase
		Ratio (percentage points)	1.6	9.5	7.9	Increase
	Place of residence (urban or rural)	Difference (percentage points)	3.5	3.8	0.3	Increase
		Ratio (percentage points)	1.3	2.58	1.28	Increase
Modern contraception use (%)	Economic status (wealth quintiles)	Difference (percentage points)	6.4	5.8	-0.6	Decrease
		Ratio (percentage points)	1.5	30	28.5	Increase
	Education (no studies, primary, secondary)	Difference (percentage points)	8.4	10.3	1.9	Increase
		Ratio (percentage points)	1.6	10.36	8.76	Increase
	Place of residence (urban or rural)	Difference (percentage points)	3.4	3.6	0.2	Increase
		Ratio (percentage points)	1.3	4.27	2.97	Increase
Adolescent fertility rate (per 1000 women ages 15–19)	Economic status (wealth quintiles)	Difference (percentage points)	32.9	9.3	-23.6	Decrease
		Ratio (percentage points)	1.4	2.26	0.86	Increase
	Education (no studies, primary, secondary)	Difference (percentage points)	122.7	9.4	-113.3	Decrease
		Ratio (percentage points)	6.7	2.49	-4.21	Decrease
	Place of residence (urban or rural)	Difference (percentage points)	34.4	2.4	-32	Decrease
		Ratio (percentage points)	1.3	1.27	-0.03	Decrease
Antenatal care (%)	Economic status (wealth quintiles)	Difference (percentage points)	4.9	44.1	39.2	Increase
		Ratio (percentage points)	16.2	4.64	-11.56	Decrease
	Education (no studies, primary, secondary)	Difference (percentage points)	5.6	50.9	45.3	Increase
		Ratio (percentage points)	5	2.97	-2.03	Decrease
	Place of residence (urban or rural)	Difference (percentage points)	3.8	14.2	10.4	Increase
		Ratio (percentage points)	8.5	1.41	-7.09	Decrease
Births delivered by a health professional (%)	Economic status (wealth quintiles)	Difference (percentage points)	66.2	54.4	-11.8	Decrease
		Ratio (percentage points)	7.2	6.61	-0.59	Decrease
	Education (no studies, primary, secondary)	Difference (percentage points)	45.5	56.6	11.1	Increase
		Ratio (percentage points)	2.7	2.14	-0.56	Decrease
	Place of residence (urban or rural)	Difference (percentage points)	50.5	14.1	-36.4	Decrease
		Ratio (percentage points)	4.5	1.38	-3.12	Decrease
Deliveries in a health facility (%)	Wealth quintile	Difference (percentage points)	30.7	42.7	12	Increase
		Ratio (percentage points)	22.9	9.5	-13.4	Decrease
	Place of residence	Difference (percentage points)	17.4	7.7	-9.7	Decrease
		Ratio (percentage points)				Decrease
		Difference (percentage points)	6.8	1.4	-5.4	Decrease
		Ratio (percentage points)				Decrease
Under-fives with diarrhoea taken to a healthcare facility (%)	Economic status (wealth quintiles)	Difference (percentage points)	8	39.1	31.1	Increase
		Ratio (percentage points)	5	3	-2	Decrease
	Education (no studies, primary, secondary)	Difference (percentage points)	9.9			
		Ratio (percentage points)	3.5			
	Place of residence (urban or rural)	Difference (percentage points)	6.2	7.7	1.5	Increase
		Ratio (percentage points)	3.2	1.1	-2.1	Decrease
Early initiation to breastfeeding	Economic status (wealth quintiles)	Difference (percentage points)	6.2	6.8	0.6	Increase
		Ratio (percentage points)	1.3	1.12	-0.18	Decrease
	Education (no studies, primary, secondary)	Difference (percentage points)	5.5	6	0.5	Increase
		Ratio (percentage points)	1.2	1.1	-0.1	Decrease
	Place of residence (urban or rural)	Difference (percentage points)	0.5	0.3	-0.2	Decrease
		Ratio (percentage points)	1	1	0	No change
Stunting prevalence in children aged < 5 years (%)	Economic status (wealth quintiles)	Difference (percentage points)	26.1	5.6	-20.5	Decrease
		Ratio (percentage points)	2	1.69	-0.31	Decrease
	Education (no studies, primary, secondary)	Difference (percentage points)	19.3			
		Ratio (percentage points)	1.7			
	Place of residence (urban or rural)	Difference (percentage points)	15.4	1	-14.4	Decrease
		Ratio (percentage points)	1.5	1.11	-0.39	Decrease
Wasting prevalence in children aged < 5 years (%)	Economic status (wealth quintiles)	Difference (percentage points)	9.9	0.9	-9	Decrease
		Ratio (percentage points)	2.5	1.17	-1.33	Decrease
	Education (no studies, primary, secondary)	Difference (percentage points)	3.4			
		Ratio (percentage points)	1.3			
	Place of residence (urban or rural)	Difference (percentage points)	7.6	3.2	-4.4	Decrease
		Ratio (percentage points)	1.9	1.42	-0.48	Decrease
Severe wasting prevalence in children aged < 5 years (%)	Economic status (wealth quintiles)	Difference (percentage points)	4	3.2	-0.8	Decrease
		Ratio (percentage points)	4.4	2.68	-1.72	Decrease
	Education (no studies, primary, secondary)	Difference (percentage points)	2.2			
		Ratio (percentage points)	2			
	Place of residence (urban or rural)		3.3	0.8	-2.5	Decrease
			2.5	1.4	-1.1	Decrease
Under-five mortality rate (per 1000 live births)	Economic status (wealth quintiles)	Difference (percentage points)	60.86	51.44	-9.42	Decrease
		Ratio (percentage points)	1.49	1.61	0.12	Increase

Between 2006 and 2018–19, the ratio of the highest-income pregnant women attending four prenatal visits decreased by 11.56% points. The ratio of women attending four prenatal care visits with secondary education to those without education decreased by 2.03% points and the ratio of urban to rural mothers decreased by 7.09% points.

### Skilled birth attendant

The percentage point difference between the rate of women from the highest-income quintile and the lowest with a midwife or skilled birth attendant present during labour increased between 2006 and 2018–19. The same was true of the ratio of these two income groups. The gap—measured as the percentage point difference — between women with secondary education and women without- increased significantly. There was a slight decrease between the ratios of the two time periods (see Table 1). The difference and ratio between urban and rural mothers giving birth attended by a health specialist decreased between 2006 and 2019.

### Rate of births in a healthcare facility

The percentage point difference between the highest and lowest income quintiles for the rate of births delivered in a healthcare facility increased between 2006 and 2019 by just over 10 points. However, the prevalence doubled during this same period (Table 1). The ratio of the 2 decreased by half. The difference and ratio by place of residence, urban to rural, decreased between 2002 and 2006 and then again between 2006 and 2019 (see Table 1).

### Early initiation of breastfeeding

The analyses of changes in inequality measures in early initiation to breastfeeding between 2006 and 2009 show very little change. For economic status, the difference in the percentage of newborns breastfed within one hour between those infants in the highest-income quintile and those in the lowest increased by 0.6% points between 2006 and 2009. The ratio between these two groups decreased by 0.18% points during the same period. Between 2006 and 2018–2019, the difference in early breastfeeding between newborns born to mothers with the highest level of education and the lowest increased by 0.5% points; the ratio decreased by 0.1. The difference in the same variable between children living in cities and children living in rural areas decreased by 0.2% points, and the ratio remained the same between 2006 and 2018–2019.

### Stunting and wasting

The difference—measured in percentage points — in the stunting prevalence of the highest-income children and the lowest decreased by 20.5 between 2006 and 2018–19. The difference in the stunting prevalence between children from urban households and rural children decreased by 14.4% points. Table 1 shows further results.

The difference in the wasting prevalence of the highest and lowest-income children decreased by 9% points between 2006 and 2018–19. The ratio of these two groups decreased by 1.33% points over the same period. The difference in the wasting prevalence between children from urban households and rural children dropped by 4.4% points. The ratio of urban to rural children under five decreased by 0.48 during the same years. The difference and ratio between income groups in the prevalence of severe wasting among children under age five also decreased between 2004 and 05 and 2018–19. The difference and ratio between urban and rural children under five decreased during these years. Table 1 shows further results.

### Care-seeking

The difference– measured as percentage points– between children of highest-income households with diarrhoea taken to a healthcare facility and the lowest increased by 31.1 between 2006 and 2018–19. The percentage point difference between children from urban households with diarrhoea being taken to see a doctor or nurse and rural ones being taken increased between the two time periods from this study. Table 1 shows further results.

### Under-five mortality

The difference in under-five mortality rates per 1,000 live births between children born to mothers with the lowest and highest incomes has decreased. In 2006, the difference between under-five mortality rates for the two groups was 60.86, with a ratio of 1.49. By 2019, the difference had decreased to 51.44 points, and the ratio was 1.61. There is an income slope in the mortality rate among children under five. The lower the income is, the higher the under-five mortality rate. Under-five mortality rates declined between 1990 and 2019 across all income quintiles. Absolute inequalities also fell from a difference of 64.1 points in 1990 to 51.4 in 2019. However, relative disparities increased. The lowest and highest income quintile ratio was 1.5 in 1990 and rose to 1.6 in 2019.

## Discussion

Our research findings indicate that mothers and children living in rural areas of Somalia with limited financial resources and low or noeducational attainment have less access to reproductive, prenatal, maternal and child healthcare as compared to their urban counterparts with higher wealth levels. Moreover, these children are more prone to malnutrition. Accessing disaggregated data for 2006 and 2018-19 permitted us to assess changes in inequalities in RMCH in Somalia over this period. The key results show that although the gap in child malnutrition and under-five mortality was reduced, the disparities in RMCH widened. Moreover, while aggregate and absolute inequalities fell, relative inequalities grew.

One of the contributing factors to the pervasive inequalities in RMCH in Somalia–reported in our findings–is the slower adoption of contraception in rural areas, which leads to higher fertility rates and increased strain on mothers and resources [20]. Community-level values and norms surrounding marriage and fertility are more entrenched in rural areas [21, 22]. These reinforce traditional reproductive ideals that are not conducive to favourable and fair health outcomes for children and their mothers [23–25]. In Somali culture, it is customary for parents to endorse their young daughters’ marriages. However, this practice not only hinders the realisation of these young girls’ full potential and development but also leads to a more extended reproductive period and higher levels of fertility, which in Somalia are among the highest worldwide (6.9 children per woman) [12]. As a result, it is imperative to consider the potential drawbacks of this traditional practice. The Somali government’s ‘Voluntary National Review Report 2022’ highlights that early and forced marriage is deeply ingrained in society and will require combined and concerted efforts to address it [26].

Deaths caused by maternal conditions are the leading cause of death among females of reproductive age in Somalia [27]. It has been amply reported that prenatal care is an important entry point into the health system for expectant mothers, and qualified health care during birth is essential for the survival of both mothers and neonates [28]. However, as our findings show, not only is the rate of access to antenatal care in Somalia extremely low, inequalities have continued to widen. A study by Yaya et al. confirms our findings regarding Somalia’s worryingly low rates of appropriate maternal care [29]. In its cross-country comparisons, Somalia is among the worst-performing countries, with Afghanistan and South Sudan. Compared with other world regions, the most significant inequality in antenatal care use was observed in sub-Saharan Africa [29]. Low coverage rates and inequalities in access to antenatal care not only hinder chances of identifying possible complications during birth, such as pre-eclampsia, but also significantly reduce the chances of delivering in a healthcare facility with trained medical staff present. An explanation for the inequalities in antenatal care and during delivery by place of residence reported in our findings is that Somalia has an entirely unreliable transport and communication systems infrastructure. Coupled with severe safety issues for women and girls, it is curtailing the chances of rural and nomadic women receiving the care they need [30]. Residential inequalities in RMCH are an issue in Somalia and other low and middle-income countries (LMIC), with rural areas bearing the brunt, as healthcare services are concentrated in urban areas [31, 32].

Female disempowerment concerning healthcare-seeking and decision-making in Somalia is a critical issue that affects women’s access to services [28]. According to the SHDS, 45% of Somali women reported that their husbands make health decisions for them, and 34% make joint decisions; 42% of women referred to this as a significant barrier to their accessing healthcare [12]. This will undoubtedly have contributed to the unequal access to RMCH services shown in our results; 43.4% of women with no educational qualifications stated that they need their husbands’ permission to seek medical assistance, compared with 18% with higher education [12]. The fact that men are still in control of decisions concerning women’s health is not only a crucial human rights and equity violation. It also significantly reduces the chances of rural, nomadic and low income women surviving labour [12, 33].

Moreover, it has been reported elsewhere that there is minimal health-related knowledge among these population groups, contributing to poor health-seeking behaviour and service demand in Somalia. This will undoubtedly compound the disempowerment of women regarding the crucial healthcare they require [34]. Programmes that improve the health literacy of men and women regarding essential healthcare for women are needed in rural and nomadic areas in Somalia and among the poorest urban households.

To overcome persistent inequalities in healthcare seeking for children, it is vital to take into account that children of mothers with higher qualifications show improved nutritional status and lower mortality [35]. Receiving and education enables mothers to make more informed decisions regarding their children’s food intake, hygiene and preventive care [36, 37]. Therefore, ensuring equal access to schooling for girls in remote areas is essential in Somalia, given that it is the mothers who are responsible for children’s health [38].

Another critical issue raised in this study is that Somali women face challenges in expressing their health concerns and symptoms to the male-dominated healthcare workforce (78.4% of health workers are male) [26, 30]. And seek medical advice from traditional sources, such as older females within the family or clan [39]. Moreover, a mixed methods report on inequalities in RMCH in Somalia showed that the health worker density in Somalia is 0.92 health workers per 1000 people [40]. This is considerably lower than WHO’s threshold for critical human resource shortages: 2.28 health workers per 1000 people [41]. Therefore, it is crucial to improve the health infrastructure in Somalia with more trained personnel and to make it more equitable gender-wise.

Furthermore, for the Somali population, financing their healthcare severely burdens households. Nomadic and rural households who must ask for borrowed money to cover medical expenditures, are among the most burdened [12]. It has been reported elsewhere that socially disadvantaged women are challenged by out-of-pocket fees for maternal health services and by the cost of transport [42]. In Somalia, income inequalities in healthcare access are compounded further by the recurring climate crises, which affect the livelihoods of rural and nomadic populations the most [26]. Thus, reinforcing and perpetuating the cycle of poverty and poor health among vulnerable households and communities.

The decline in absolute inequalities in the U5MR was significant, thus showing that as the overall mortality levels decreased, absolute inequalities also fell. The decrease in inequalities in malnutrition demonstrated in our findings will have contributed to these reductions in absolute inequalities in under-five mortality, even though inequalities in access to care for children have increased. However, because the absolute U5MR remains high, it might explain why relative inequalities increased slightly. Previous research suggests that relative disparities may continue to grow as national-level under-5 mortality rates further decrease [43]. This is consistent with findings from another similar study by Chao et al.; between 1990 and 2016, absolute inequalities in the U5MR decreased notably across all world regions, whereas relative inequalities increased in eastern and southern Africa. However, these remained similar in most other low and middle-income countries. To reduce relative and absolute inequalities, interventions must substantially increase their reach and effectiveness by targeting children from the bottom wealth quintile.

## Study limitations

Despite the scarcity of information and reliable estimates for health and nutrition indicators in Somalia, we have evaluated the progress in reducing disparities in maternal and newborn health in the country between 2006 and 2019–19 using available data from secondary sources. The scarcity of reliable data in Somalia goes hand in hand with the fact that only 6% of children under five were registered in 2019 [44]. These data gaps present severe challenges to monitoring inequality and establishing trends in Somalia’s child and maternal health inequalities [45]. Even compared to other low- and middle-income countries, Somalia’s health data are severely limited. We accessed health and determinants data from the only publicly available sources that provide disaggregated data based on economic position, education, and place of residence in Somalia.

We chose our two data sources based on their reliable methodology and the experienced staff who collected and ensured the data quality [13]. We used secondary data from 2 different surveys, which enabled us to compare two time points to establish changes in inequalities in RMCH in Somalia. The data acquired from the SDHS do not provide confidence intervals (CI), even though the data from MICS 2006 do. Therefore, we were unable to report the CI in our results, which may have negatively affected their interpretation..

The data accessed from the MICS 2006 and the SDHS 2020 are only disaggregated by one dimension of inequality at a time; therefore, we were not able to establish whether mothers and children from low-income households had higher rates of access to healthcare depending on whether they lived in an urban or rural area or depending on whether the mother had received some education.

The simple summary measures computed for this study do not consider values in the other subgroups when there are over two groups, for wealth quintiles and educational level. We could not produce summary measures for inequality distribution across all wealth quintiles or qualification levels, only for the highest and lowest ones. Also, the nature of the simple measures employed in these analyses cannot account for the population size of each group as these are unweighted and compute all the groups equally. Complex measures avoid such limitations; however, the reduced availability of data in Somalia, especially disaggregated data, meant that we could only use simple measures in our analyses [46].

## Conclusions

Between 2006 and 2019–19, progress was made in Somalia in reducing inequalities in malnutrition, a positive development in Somalia’s public health landscape, and has likely contributed to reducing inequities in the under-five mortality rate (U5MR). However, inequalities in access to contraception and essential healthcare for expecting mothers have increased over the same period, as well as care-seeking for sick children under five.

Improving the health literacy of men and women in Somalia with a gender-equity perspective is imperative, especially among rural populations and remote communities. There is an urgent need to intensify efforts to provide women and children with the essential healthcare needed for survival and healthy development. Adequate additional resources that are accessible to all mothers and their children, especially rural women, are desperately needed. These include qualified female medical staff and equipment.

## Data Availability

The datasets used and analysed during the current study are available from the corresponding author upon request.
